# Access to Guideline-Recommended Pharmacogenomic Tests for Cancer Treatments: Experience of Providers and Patients

**DOI:** 10.3390/jpm7040017

**Published:** 2017-11-15

**Authors:** Ann Chen Wu, Kathleen M. Mazor, Rachel Ceccarelli, Stephanie Loomer, Christine Y. Lu

**Affiliations:** 1 Precision Medicine Translational Research (PROMoTeR) Center, Department of Population Medicine, Harvard Pilgrim Health Care Institute and Harvard Medical School, 401 Park Drive, Suite 401, Boston, MA 02215, USA; rceccare@bu.edu (R.C.); stephanie_loomer@harvardpilgrim.org (S.L.); christine_lu@harvardpilgrim.org (C.Y.L.); 2 Meyers Primary Care Institute, A Joint Endeavor of the University of Massachusetts Medical School, Reliant Medical Group and Fallon Health; 630 Plantation Street, Worcester, MA 02215, USA; Kathy.Mazor@meyersprimary.org

**Keywords:** pharmacogenomic tests, cancer, access, providers, patients

## Abstract

Genomic tests are the fastest growing sector in medicine and medical science, yet there remains a dearth of research on access to pharmacogenomic tests and medications. The objective of this study is to explore providers’ and patients’ experiences and views on test access as well as strategies used for gaining access. We interviewed clinicians who prescribed medications that should be guided by pharmacogenomic testing and patients who received those prescriptions. We organized the themes into the four dimensions suggested by the World Health Organization framework on access to medications and health technologies. Guideline-recommended pharmacogenomic tests for cancer care are generally available, although the timeliness of return of test results is sometimes suboptimal. Accessibility of pharmacogenomic tests is made challenging by the process of ordering pharmacogenomic tests, which is time-consuming. Affordability is a barrier to some patients as expressed by both providers and patients, who noted that the cost of pharmacogenomic tests and medications is high. Acceptability of the tests is high as both providers and patients view the tests positively. Understanding challenges to accessing pharmacogenomic tests will allow policymakers to develop policies that streamline access to genomics-based technologies to improve population health.

## 1. Introduction

Precision medicine and genomics-based technologies promise safer and more effective use of pharmacotherapy. Pharmacogenomic tests can predict which individuals are at risk of toxic response to a drug, thereby minimizing drug-related adverse events and the associated costly consequences, such as hospital admissions [1,2]. Such tests can also identify individuals likely to respond to an intervention based on molecular markers. As of November 2016, there were over 48,000 genetic tests for 10,000 conditions and 4200 genes [3]. The Centers for Disease Control and Prevention (CDC) estimates two to three new genomic tests each week [4]. Recommendations from the Food and Drug Administration (FDA), such as the information included in drug labels, the CDC, and national professional organizations are available to aid clinical decision-making around use of pharmacogenomic tests based on evidence [5,6,7,8]. 

Precision medicine is particularly advanced in oncology; pharmacogenomic tests guiding cancer treatments account for over 80% of genomic tests with sufficient evidence for clinical utility, and clinical and analytical validity [9]. Tumor markers have dramatically changed the treatment of cancer in the last decade with many available pharmacogenomic tests and the promise of many advancements to follow [10]. Guidelines currently recommend pharmacogenomic testing to inform decision making about medications for many types of cancers because there is sufficient evidence of benefits [11,12,13]. For example, human epidermal growth factor receptor 2 (HER2) predicts benefit or resistance to anti-HER2 therapies such as trastuzumab for breast cancer [14]. Differences in access to guideline-recommended pharmacogenomic tests may affect subsequent treatment decisions and ultimately impact patients’ health outcomes [15]. Studies report that providers’ rates of ordering pharmacogenomic tests vary [16], but the reasons for such variations are not well understood. Information on the barriers and facilitators of access to guideline-recommended pharmacogenomic tests could provide insights into these variations and help to enhance appropriate access. The objective of this qualitative study was to explore providers’ and patients’ experiences in access to pharmacogenomic tests and medications in the context of cancer care. 

## 2. Methods

### 2.1. Overall Design

This descriptive study, using semi-structured qualitative interviews to explore the experiences and views of patients and providers, was approved by the Institutional Review Board at Harvard Pilgrim Health Care (HPHC; Project ID 922106-1). Provider and patient participants were offered a $25 gift card in appreciation for their time.

### 2.2. Sampling

Potential participants were identified through insurance claims data from HPHC, a regional health plan covering about 1 million members from New England. We identified 126 providers who had prescribed any of a selected list of cancer targeted therapies from 1 May 2013 to 30 April 2014 using HPHC claims data; the prescription of these agents could be informed by pharmacogenomic tests in accordance with clinical guidelines (Table 1). Based on HPHC claims data from 1 May 2014–30 April 2015, we identified 261 patients who had been prescribed any of a selected list of cancer targeted therapies; of these 18 were on the ‘do not contact’ or deceased list and 13 were missing contact information. 

We mailed invitation letters to 126 providers and 230 patients, asking them to call a toll-free line or email if they were interested in participating in a telephone interview. Based on our early interviews with providers, we learned that in some locations, ordering tumor biomarker and pharmacogenomic tests and insurance billing involves other healthcare professionals (i.e., surgeons, pathologists, genetic counselors, nurse managers, patient navigators). Because our goal was to examine access to pharmacogenomic tests and barriers to access, we modified our recruitment approach for providers and allowed snowball sampling to interview the extended range of abovementioned healthcare professionals if our oncologist interviewees indicated other healthcare professionals were involved in the routine process of accessing pharmacogenomic tests/genetic tests in their practice. To obtain diverse views, we purposively sampled clinicians in community and academic settings.

### 2.3. Interview Process and Data Collection

Provider and patient interviews were conducted via telephone by one of two study investigators, Wu and Ceccarelli. Interview questions explored concepts specified in the World Health Organization (WHO) framework to govern access to medicines [17]. The framework includes the following components: (1) availability, which includes types and quantity of technology (in the case of pharmacogenomic tests, availability includes test results); (2) accessibility, which refers to physical access to the technology; (3) affordability, which includes out of pocket burden; and (4) acceptability, which includes patient and provider attitudes towards and expectations of products and services including patient-provider communication about acceptability. 

In conducting the interviews, the questions served as a guide, and the interviewer had discretion in phrasing, using probes, and posing additional questions. Interviews lasted approximately 30 min. We conducted interviews until no new themes emerged, which occurred once we interviewed ten clinicians and sixteen patients.

### 2.4. Analysis

The initial coding scheme was developed by the study team using the interview questions as an initial organizing framework. Two investigators then independently read 26 transcripts (10 providers and 16 patients), and generated specific codes to capture relevant content within each of the major thematic areas (e.g., affordability). Provider transcripts were analyzed by Wu and Ceccarelli, and patient transcripts were analyzed by Wu and Loomer; other study investigators contributed to the interpretation of results. Provider and patient codes were generated separately. We followed the phases of thematic analysis as described by Braun and Clarke [18]. First, we familiarized ourselves with the data by transcribing the data, reading and re-reading the data, and noting down initial ideas. Second, we generated initial codes. Third, we searched for themes, while collating codes into potential themes, gathering all data relevant to each potential theme. Fourth, we reviewed the themes. Fifth, we defined and named the themes and categorized them according to the domains of the WHO framework. Sixth, we produced the report and selected examples of compelling extract examples to produce this report. 

## 3. Results

Of the ten clinicians who participated, eight were oncologists and two were nurse practitioners; seven were male, five were ages 40–49 years, four were 50–59 years, and one was 30–39 years. Six of the clinicians were Caucasian, three were Asian, and one was Caucasian/Hispanic. Sixteen patients were interviewed with one age 30–39 years, one age 40–49 years, seven age 50–59 years, and seven over 60 years. Nine of the patients interviewed were female and seven were male. Patients were diagnosed with different types of cancer including breast (5), colorectal (1), non-small cell lung cancer (4), and leukemia (4) (Table 2).

### 3.1. Overview

Table 3 highlights key quotes from the interviews by themes in this study. We categorized the themes into the four dimensions of access to medicines defined by the WHO framework [17].

### 3.2. Availability

#### 3.2.1. Provider Interviews

Some providers reported concerns with the timeliness of availability of test results as the results of pharmacogenomic tests can take a while to return and be too late to help clinically. One provider mentioned, “We’ve had two patients whose disease was at a galloping pace and unfortunately they died from their disease before we could get the results of the genetic testing … where it did reveal that they had a mutation”.

#### 3.2.2. Patient Interviews

Patients also reported concerns with tests taking a long time to come back. One patient stated, “He (the physician) has to negotiate with (the hospital) to get a piece of tissue to send to him. He literally said that would take a few weeks and then the process of payment and all that”. Other patients acknowledged that the process of obtaining pharmacogenomic testing could take a while as one patient stated, “He literally said that (the test) would take a few weeks and then the process of payment and all that. I’d say it’s a process”. 

### 3.3. Accessibility

#### 3.3.1. Provider Interviews

Some providers did not report encountering barriers with accessing pharmacogenomic tests. One provider stated, “I think nowadays in 2015 it’s pretty much straight forward. I think we’ve bypassed the barrier phase. But since I was here and I had to go to the process of implementing the idea that the testing is important, I can tell you that the picture was very different even five or seven years ago”. Other providers reported that variability and inefficiency in the process of pharmacogenomic testing led to decreased accessibility. The process of ordering pharmacogenomic tests is not standardized across institutions. Providers noted that many clinicians, including the physician who conducted the biopsy, the surgeon, pulmonologist, medical oncologist, or pathologist, can order the pharmacogenomic tests. One provider stated, “In our hospital … We have determined that any provider in the nodal point of care for the patient can order … We don’t have a one defined way to do it. I must say that in reality … seventy five percent of the time, the medical oncologist will place the order for the test”.

Another potential barrier to access is navigating and dealing with patients’ insurance coverage, which can be complicated and time consuming. One provider declared, “You see so many different patients with so many different insurance plans and different prescription plans and you don’t know what’s covered, what’s not and a large part of our day is spent figuring out what’s covered, what’s not, what needs a prior authorization, what doesn’t, what the insurance will cover”. In response to the time-consuming process of dealing with insurance, some providers avoid dealing directly with insurance companies and use third party vendors who serve as a link between providers and insurance companies. Providers noted that prior authorization was not required for pharmacogenomic tests at the time of the interviews, but anticipated that this would likely change soon. This could add complexity to the current workflow and could be time-consuming and an administrative burden. 

#### 3.3.2. Patient Interviews

Few patients expressed concerns relating to administrative barriers. Some received testing without knowing the reasons for testing and some did not recall having pharmacogenomics tests in particular due to the large number of tests they underwent. For example, when asked about their experience with pharmacogenomic testing, one patient stated, “No, I wouldn’t remember if they had tested that. There was a lot of, a lot of tests I went through that week in the hospital”. Almost all patients voiced concerns and/or frustration relating to navigating insurance issues related to cancer drugs. One patient expressed, “No, no there really wasn’t a lot of talk on the insurance. I had to do all my own leg work … when you’re trying to go through your cancer treatments and try to figure out insurance, it’s like a part time job and it’s mind boggling”. In contrast, some patients received assistance and support from nurses and social workers for the paperwork. One patient stated, “So he helped us to fill out a form that would help possibly to defer the cost”. 

### 3.4. Affordability

#### 3.4.1. Provider Interviews

Most providers thought that pharmacogenomic testing is affordable to patients with insurance with some exceptions. One provider stated, “I haven’t seen refusal for any of these tests. But sometimes the patient does get a big bill, and we’ll have to then go back to the record and support the reason why it was ordered. The majority of times we are not asked to justify anything before ordering the test”. Providers noted that the “newer tests or these very large genetic panels” are less likely to be covered. In some instances when insurance does not cover the pharmacogenomic test, the hospital absorbs the cost of testing according to one provider who said, “But for the cases in which the test is not reimbursed, I believe our institution eats up the cost”. Another provider indicated that patients pay or forego the test totally, saying, “The patient either pays out of pocket, tries to get funding from another source, or just doesn’t have the test done”. In some instances, insurers may have preferences for certain laboratories, thus leading to economic barriers for patients. As one provider stated, sometimes after pharmacogenomic tests are done, “few patients … had large bills sent to them because they were told by the insurance they can only go to certain labs to get those tests done”. 

#### 3.4.2. Patient Interviews

Many patients cited issues with a lack of information about pharmacogenomic testing to them. Patients’ experience with affordability of pharmacogenomic tests varied. Some patients found the test affordable because their insurer covered the test; one patient stated, “We didn’t have to pay anything for the ‘pharmacogenomic test’. I’ve had (insurer) for a long time. Everything was covered”. On the contrary, some patients found the patient cost-sharing structure to be confusing with unclear and non-transparent information and out-of-pocket costs to be very high. One patient said, “I think that was probably my most disappointing piece of the whole cancer journey was that I really didn’t get a lot of that support to say this is how much out of pocket cost it’s going to be for you. I think, you know, if they were more upfront about it, and I know different hospitals charge different rates, so it was really difficult to get any real answers as to what things were going to actually cost and it was very difficult”. Some patients stated they were only made aware of their out-of-pocket costs when they received the bill. Although the focus of the interviews was on pharmacogenomic tests, patients largely expressed major concerns about the high out-of-pocket costs related to cancer treatment. One patient expressed, “I would get receipts from (insurer) explaining what the bills were and how they took care of them and the amounts of the bills and what they were for. The bills seemed exorbitant to me”.

Patients reported using a variety of strategies to manage costs. These included: paying out of pocket, forgoing the test (or drug), shopping for insurers, joining a clinical trial, obtaining financial assistance (e.g., through the hospital), and obtaining the medication in another state. One patient stated, “we went down and interviewed all the insurance companies because they were going to drop (insured patient) because of cost”. Some patients felt that they had no option but to pay for testing and one patient stated “So he said not all insurances cover it, he let us know that it could cost about $5000–6000 … So we said well yeah $5000–6000 in the scheme of things when you’re thinking about your life, … we’ll figure it out”.

### 3.5. Acceptability

All of the interviewees—patients or providers—expressed that pharmacogenomic testing was an asset to clinical practice.

### 3.6. Provider Interviews

In general, providers recognized the need and value of testing in clinical practice. One provider stated, “For example, for diffuse large B-cell lymphoma, there is information now that the presence or absence of certain genetic translocations can have an impact on prognosis and potentially affect treatment”. Providers have been satisfied with the process of ordering tests with one provider stating, “I think nowadays it’s pretty much straight forward. I think we’ve bypassed the barrier phase”.

### 3.7. Patient Interviews

The acceptability of pharmacogenomic tests was high. Patients recognized that test results are informative, as subtypes of cancers might be associated with treatment response and improved quality of life. One patient stated, “They said there was better quality of life if there is a mutation and I can go on a targeted therapy that would be much more preferred treatment as opposed to going through infusion chemotherapy treatment”. Another stated, “Yeah, Herceptin is hugely helpful. Every woman that has HER2 needs to have it, no questions asked”. Patients also noted that testing could confirm their eligibility and insurers would then cover expensive medicines as one patient said, “They said most of the insurance companies would cover (Tarceva) based on having the biopsy and the identification of the mutation”.

## 4. Discussion

Our study, which examines challenges to access to guideline-recommended pharmacogenomic tests and medications for cancer from the point of view of providers and patients in the current health care system, has four key findings. First, guideline-recommended pharmacogenomic tests for cancer care are generally available, although the timeliness of return of test results is sometimes suboptimal and may impede care. Second, accessibility to pharmacogenomic tests is made challenging by the process of ordering pharmacogenomic tests, which is time-consuming and complex for providers. These issues are even more prominent for multi-gene tests. As prior authorizations become required for pharmacogenomic tests, this process is likely to become more complicated and time consuming. Third, affordability is a barrier to at least some patients as expressed by both providers and patients who noted the high cost of pharmacogenomic tests and medications. There is variability in how testing is paid for. The cost of testing might be paid for by insurers, by patients who pay out of pocket, or by the hospitals. Sometimes, patients may forego the test. Fourth, acceptability of the tests is high as both providers and patients view the tests positively, and feel that patients benefit greatly from targeted therapies. However, overall it appears that patients are receiving guideline-recommended cancer pharmacogenomics tests.

Many providers in our study are concerned that the introduction of prior authorizations for pharmacogenomic tests in the foreseeable future will decrease the accessibility of such tests. This concern is based on their experience with prior authorization requirements for cancer drugs. Providers in our study voiced the desire for prior authorization procedures to be streamlined, which is consistent with prior studies [19]. The goal of prior authorization from the insurer’s perspective is to manage utilization, namely, to ensure that only patients who need certain medications actually get them. However, the prior authorization process is typically lengthy and can be costly, whether completed by physicians, nurses, or other staff [20]. In the medical literature, research suggests that the prior authorization process impacts the quality and continuity of care among the mentally ill, including forgoing treatment initiation and accelerating treatment discontinuation [21]. 

Our study reveals concerns from providers that costs may be a barrier to patients for accessing guideline-recommended pharmacogenomic tests when they are part of a gene panel test. It appears that the economic barriers experienced are minimal when these are available as single gene tests. The effects of the high cost of gene panel tests has not been reported in the literature, yet may become similar to the economic barriers relating to cancer drugs, which have also received substantial attention in the media and the literature [22]. Neumann et al. reported that 84% of 787 oncologists surveyed indicated that patients’ out-of-pocket costs influence their treatment recommendations [23]. Meisenberg et al. reported that 47% of 132 patients in a survey were concerned about financial stress related to the costs of cancer treatment [24]. Economic barriers are likely to impact patients’ access to care, care-seeking behaviors, and subsequently health outcomes [22,25,26]. Although not presented in this manuscript, providers and patients in our study voiced substantial concerns about the high cost of cancer medications.

While previous studies have not studied the effects of high cost pharmacogenomics tests because they are relatively new, we compared our results to studies on the high cost of medications for cancer. Consistent with the literature on the high cost of medications, our study indicates that communications between patients and providers could be improved. Meisenberg et al. reported that 71% of 132 patients in a survey indicating they had no communication with their oncologists about costs and only 31% were informed about costs prior to treatment [27]. Studies have reported that most physicians (ca. 70%) failed to recognize the potential financial concerns of their patients and are uncomfortable or lack time to discuss such issues. We also identified several strategies that patients used for managing costs. They included: paying out of pocket, forgoing the test (or drug), insurer shopping, joining a clinical trial, obtaining financial assistance, and obtaining the technology (drug in this patient case) in another state. Cost-saving strategies reported in the literature (predominantly focused on drugs) [28] included: copay assistance, free samples, changing/adding insurance plans, changing logistics of care (e.g., switching to lower-cost generic drug or alternative therapy/diagnostic, changing dosage/frequency, or stopping interventions). 

To our knowledge no studies have addressed the perspectives of providers and patients on experienced or perceived challenges to accessing guideline-recommended pharmacogenomic tests, and our study helps fill this gap. Nevertheless, the limitations to our study deserve mention. First, during the interviews, multi-gene tests were only emerging in clinic practice. Thus, our findings may not be generalizable to access issues related to multi-gene tests containing tumor biomarkers and other types of genomic technologies. Second, at the time of this study, prior authorizations were not commonly required by insurers for pharmacogenomic tests, although multiple providers expected this would occur soon. Many concerns expressed were specifically related to prior authorizations for cancer medications. But prior authorization requirements and processes are likely similarly burdensome for pharmacogenomic tests when they are commonly implemented. Furthermore, because we selected patients who had received cancer medications that should be informed by pharmacogenomic tests, we may have missed issues that deter people from getting the tests and the medications altogether. Because we focused on an insured population, access issues due to lack of insurance were not explored in this study. Moreover, the number of interviews was relatively small which may limit the generalizability of the findings; nevertheless, we conducted interviews until no new themes emerged. 

## 5. Conclusions

Overall, it is reassuring that there seemed to be no substantial access issues related to guideline-recommended cancer pharmacogenomic tests when they are available as single gene tests. As pharmacogenomic tests demonstrate clinical value, timely and affordable access is important for patient care. Nevertheless, we identified some concerns related to availability, accessibility, and affordability of guideline-recommended pharmacogenomics tests. Addressing these challenges is critical to improving access to and affordability of pharmacogenomic tests, in order to inform treatments. In addition, our study shows early concerns from providers that access might be a barrier to patients when pharmacogenomic tests are part of a gene panel test. Research is needed to investigate access issues related to multi-gene tests. 

## Figures and Tables

**Table 1:** Study drugs and pharmacogenomic tests.

Drugs	Gene	Examples of Test
Cetuximab	*KRAS*	Therascreen^®^ KRAS RGQ PCR Kit (Qiagen, Hilden, Germany)
Panitumumab		
Trastuzumab	*HER2*	PathVysion™ HER-2 DNA Probe Kit (Abbott Molecular Abbott Park, IL, USA); HercepTest™ (Agilent, Santa Clara, CA, USA)
Pertuzumab		
Ado-trastuzumab emtansine		
Lapatinib		
Trametinib	*BRAF*	THxID^®^-BRAF (bioMérieux, Cambridge, MA, USA)
Dabrafenib		
Vemurafenib		
Cetuximab	*EGFR*	EGFR pharmDx™ (Agilent); cobas^®^ EGFR Mutation Test (Roche Molecular Diagnostics, Pleasanton, CA, USA)
Panitumumab		
Afatinib		
Erlotinib		
Panitumumab		
Crizotinib	*ALK*	Vysis ALK Break Apart FISH Probe Kit (Abbott Molecular)
Dasatinib	Philadelphia chromosome	
Imatinib		
Bosutinib		
Nilotinib		
Imatinib	c-Kit protein	c-Kit pharmDx™ (Agilent)
Imatinib	*PDGFR*	
Toxitumomab	*CD20*	

KRAS (K-Ras); HER2 (human epidermal growth factor receptor 2); BRAF (B-Raf); EGFR (epidermal growth factor receptor); ALK (anaplastic lymphoma kinase); PDGFR (platelet derived growth factor receptor); CD20.

**Table 2:** Demographics.

	Oncologists and Oncology Nurse Practioners (*n* = 10)	Patients (*n* = 16)
Age		
30–39 years	1	1
40–49 years	5	1
50–59 years	4	7
Over 60 years	0	7
Gender		
Female	7	9
Male	3	7
Race		
Asian	3	0
White	6	16
More than one race	1	0
Practice Type		
Hospital practice	6	
Community Practice	4	
Cancer Types Discussed *		
Breast	2	5
Colorectal	3	1
Non-small cell lung cancer	5	4
Leukemia	3	4
All cancers	1	0
Other	3	4

* More than one cancer type could be discussed.

**Table 3:** Themes and quotations of provider and patient interviews.

Domain	Theme	Provider Quotations	Patient Quotations
Availability	Process of pharmacogenomic tests can take long time (time for sample processing, reimbursement)	“*Two to three weeks sometimes is a long time to wait to get results back*”.	“*Well, they took the biopsy they had from the surgery and they’re sending it to a lab. I know it was sent to the lab, but he said it takes a while…*”
Accessibility	No barriers for single gene tests experienced	“*I don’t really have to work with that at all. I just submit a request and it’s usually done*”.	“*It just seemed like everything kind of flowed and it’s kind of what they do and this is the procedure and this is the process*”.
	No set systems in place; process is inefficient	“*In our hospital, there has been an evolving change on who orders the biomarkers. So that’s part of the committee that I led to make a determination for this*”.	
	Ordering is complex	“*So I don’t know, I don’t know if there’s a way to streamline that and know, you know what under a certain prescription plan or insurance plan will be covered, will be not. That we have some way of knowing this, or some way of figuring that out beforehand rather than putting the patients through all of this you know. They’re dealing with a very difficult diagnosis and it’s so stressful as it is and then to you know have patients be told well you know your insurance might not cover this or we have to find an alternative is really tough to tell people as a provider. You know it’s really, really difficult*”.	“*I know different hospitals charge different rates, so it was really difficult to get any real answers as to what things were going to actually cost and it was very difficult*”.
	Number and variety of insurance plans across patients that providers must deal with	“*So the co-payments can go anywhere from one dollar or zero to as high as in the high thousands….and I don’t know if myself or my colleagues understand necessarily who will be the person that will have a higher or lower copay*”.	
		*I don’t think they (doctors) should be expected to go to looking for whether the insurance covers something because it’d be too much work.*	
	Coverage policies are constantly changing, Prior authorization is expected	“*Five years ago, we didn’t need to get as many prior authorization for things as we do present day. Nothing has been stopped in terms of us submitting a request for (genetic testing) because of the need for a prior authorization. So it hasn’t hit as yet if it’s, if that’s what needs to be done*”.	“*In 2007, they sent off my tumor and I don’t remember signing anything. There was no discussion of who was going to pay for this. Since that time, I have had several other genetic tests and I’ve gotten mountains of paperwork. I mean 20–30 pages to sign and it’s very clear who is paying for it*”.
Affordability	Few economic barriers for tests	“*I haven’t seen refusal for any of these tests.*	“*I wasn’t aware of (insurance coverage issues). If there was, they did it behind the scenes*”.
	Certain labs approved by insurer; others are not	“*I think more of an issue has been few patients who have had large bills sent to them because they were told by the insurance they can only go to certain labs to get those tests done*”.	
	Institution absorbs cost	*For our solid tumor group, they have been sending some of these panels that involve essentially looking at mutations in a whole list of genes and the company that they have been sending the tests to kind of already have an agreement that the insurance company doesn’t pay for it, the test, the lab itself will cover the cost so the institution doesn’t get stuck with the bill.*	
	High costs of pharmacogenomic tests for insurers		“*When I met with that doctor, she said that sometimes it takes a while because insurances don’t always cover it, and my husband and I said we didn’t care because it was going to offer all the information, kind of the end point information that was necessary in order to be able to do a test drug. I guess (my insurer) is one of the insurances that were beginning to think about wanting to cover the cost*”.
	Patients can have high out of pocket costs	“*I’ve had patients pay for it because they just wanted to get it done and they were willing to pay out of pocket but most people, the cost is a big deterrent*”.	“*It’s cheaper to die than to live because you don’t want to burden your family. I’ve actually heard people talk like that and make those decisions. It’s very scary*”.
	Genetic panels are more difficult to get covered	“*I think that the major concern today is with these large genomic profile panels that there may be some issues with*”.	
	No option but to pay for testing out of pocket		“*I didn’t really have much choice. It’s not like I wasn’t going to get the test and I certainly wasn’t going to shop around for a cheaper test*”.
Acceptability	Testing is needed and valued in clinical practice	“*In today’s day and age we actually have drugs that target, that are actually useful in that situation so finding that information today would actually affect how we treat a patient*”.	“*So I did the genetic test, it said I was a good candidate for Tarceva. And that was one of the better decisions I’ve ever made*”.
	Satisfied, no issues	“*I haven’t seen refusal for any of these tests. But sometimes the patient does get a big bill, and we’ll have to then go back to the record and support the reason why it was ordered. But the majority of times we are not asked to justify anything before ordering the test*”.	“*I didn’t feel like there was an option not to get the bone biopsy. That was an imperative to get the bone biopsy in order to understand the mutations*”.
	Getting testing would help with payment of medications		“*I believe that the reason for the biopsy was so that (the hospital) could you know attest to it, the fact that Tarceva was going to benefit me and I think they, I believe they had to communicate that to (my insurer)*”.
